# Methodology for the Randomised Injecting Opioid Treatment Trial (RIOTT): evaluating injectable methadone and injectable heroin treatment versus optimised oral methadone treatment in the UK

**DOI:** 10.1186/1477-7517-3-28

**Published:** 2006-09-27

**Authors:** Nicholas Lintzeris, John Strang, Nicola Metrebian, Sarah Byford, Christopher Hallam, Sally Lee, Deborah Zador, RIOTT Group

**Affiliations:** 1 Institute of Psychiatry, King's College London, 16 De Crespigny Park, London, SE5 8AF, UK; 2 The Alliance, Room 312 Panther House, 38 Mount Pleasant, London, WC1X 0AN, UK; 3 National Drug and Alcohol Research Centre, University of New South Wales, Sydney, 2052, Australia; 4 South London and Maudsley NHS Trust. Denmark Hill, London, SE5 8AF, UK

## Abstract

Whilst unsupervised injectable methadone and diamorphine treatment has been part of the British treatment system for decades, the numbers receiving injectable opioid treatment (IOT) has been steadily diminishing in recent years. In contrast, there has been a recent expansion of supervised injectable diamorphine programs under trial conditions in a number of European and North American cities, although the evidence regarding the safety, efficacy and cost effectiveness of this treatment approach remains equivocal. Recent British clinical guidance indicates that IOT should be a second-line treatment for those patients in high-quality oral methadone treatment who continue to regularly inject heroin, and that treatment be initiated in newly-developed supervised injecting clinics.

The Randomised Injectable Opioid Treatment Trial (RIOTT) is a multisite, prospective open-label randomised controlled trial (RCT) examining the role of treatment with injected opioids (methadone and heroin) for the management of heroin dependence in patients not responding to conventional substitution treatment. Specifically, the study examines whether efforts should be made to optimise methadone treatment for such patients (e.g. regular attendance, supervised dosing, high oral doses, access to psychosocial services), or whether such patients should be treated with injected methadone or heroin.

Eligible patients (in oral substitution treatment and injecting illicit heroin on a regular basis) are randomised to one of three conditions: (1) optimized oral methadone treatment (Control group); (2) injected methadone treatment; or (3) injected heroin treatment (with access to oral methadone doses). Subjects are followed up for 6-months, with between-group comparisons on an intention-to-treat basis across a range of outcome measures. The primary outcome is the proportion of patients who discontinue regular illicit heroin use (operationalised as providing >50% urine drug screens negative for markers of illicit heroin in months 4 to 6). Secondary outcomes include measures of other drug use, injecting practices, health and psychosocial functioning, criminal activity, patient satisfaction and incremental cost effectiveness. The study aims to recruit 150 subjects, with 50 patients per group, and is to be conducted in supervised injecting clinics across England.

## Background

### The British system of injectable opioid treatment

Conventional approaches to maintenance substitution treatment using oral methadone are effective for most heroin users entering treatment (see [1] for review). However, there are a proportion of patients who do not benefit from such approaches – up to 50% of patients drop out of maintenance treatment within 12 months, and of those who remain in treatment, a substantial minority (up to 15% of most programs) continue to inject heroin on a regular basis (e.g. daily), and continue to experience considerable drug related harm [2,3].

One response for those individuals who fail to benefit from conventional substitution treatment has been the prescription of injectable opioids – methadone and diamorphine (pharmaceutical heroin). This has been a distinctive, yet dwindling feature of the 'British system' over the past 40 years [4]. The majority of patients treated with injectable opioids have received injectable methadone ampoules, and much smaller proportions have received injectable diamorphine (approximately 90% and 10% of injectable prescriptions in 1995 respectively [5]). Unlike recent developments in Switzerland, Germany, Spain and the Netherlands, where treatment centres have been established to deliver supervised injectable opioid treatment (IOT), the British system has had limited capacity for supervised dosing. The majority of IOT patients receive daily to weekly supplies of methadone or diamorphine ampoules from clinics or community pharmacies for take home unsupervised consumption [6].

However, in the past decade, the role of IOT for heroin dependence has been steadily diminishing in Britain. Injected methadone ampoules accounted for 8.7% of NHS opioid prescriptions for heroin dependence in England and Wales in 1995, but only 1.9% in 2003 [4]. In 1995, diamorphine accounted for two per cent of all opioid prescriptions for opiate dependence [5], and by 2000 this had fallen to approximately 1% [6]. Whilst exact numbers are unavailable, we estimate that in 2006, there are between 2,000 to 3,000 patients prescribed injectable methadone ampoules, and up to 500 patients prescribed diamorphine in the NHS for opioid dependence. This total number in IOT has remained relatively static over the past decade, with little 'turn-over' and few new patients commencing IOT. Yet during this same period there has been a marked expansion of numbers in opioid substitution (largely oral methadone and buprenorphine) treatment in Britain, from fewer than 50,000 to over 100,000, reflecting both a probable increase in the number of heroin users and the proportion in treatment. Thus, whilst the number of heroin dependent users and the number of patients entering substitution treatment in Britain appears to be steadily increasing over the past decade, the role of IOT has proportionally diminished, with few new patients commencing this form of treatment. The diminishing role of IOT in the UK may be due to a number of factors [6,7]:

- Limited evidence supporting IOT – the past decade has seen an increasing emphasis within modern health systems that clinical activity be based upon the principles of evidence-based practice [8,9]. Further, there is general consensus as to the quality of evidence required to establish such an evidence base [10,11]. Whilst it is possible to identify individuals or groups of clients who have been successfully treated with IOT, this does not provide sufficient evidence to establish the safety and efficacy of this treatment approach. Adequately controlled trials, comparing IOT to treatment approaches considered 'gold standard' for this patient population are required. Whilst trials of heroin treatment conducted in Switzerland [12,13] and the Netherlands [14] provide useful information on the potential benefits of prescribing heroin, the differences between the trial designs and treatment context of these trials make it unclear how far these results can be applied to a UK setting. Here in the UK, only one RCT of 96 subjects conducted in the 1970's has compared unsupervised IOT (diamorphine) to oral methadone ([15]- reviewed below). In contrast to the limited evidence base of IOT, there has been an ever increasing body of evidence supporting other substitution approaches, such as oral methadone and sublingual buprenorphine treatment.

- Concerns regarding diversion of medication – programs with low levels of supervision may be less expensive to deliver, however, widespread proliferation of treatment programs without the capacity for supervision (even of 'unstable' patients) are likely to be associated with diversion of some medication onto the 'illicit market'. Twelve percent of patients in the Hartnoll trial [15] self-reported selling part of their take away diamorphine ampoules. In a recent survey of 192 opioid dependent patients entering treatment in south London, approximately 20% reported having ever used illicitly obtained injectable methadone or diamorphine [16]. Doctors concerns over the possibility of diversion of prescriptions to others has led to a reluctance by some to prescribe injectable treatments [6]. Concerns regarding diversion and poor adherence may also limit the doses prescribed within IOT, which may in turn reduce treatment effectiveness.

- Concerns that the provision of injectables may prolong the drug use and injecting 'careers' of patients, with similar concerns that it is difficult to 'move' patients onto more conventional treatment approaches once they have been exposed to prescribed injectable opioids [15,17]. This can also result in 'silting up' of limited treatment places, restricting its availability to new patients. This is particularly relevant to the British system where the routine availability of injectable take-aways for unsupervised consumption at home may remove incentives for clients to move onto oral methadone programs.

- High cost – IOT is considerably more expensive than oral methadone treatment. The main additional costs are medication-related (e.g. approximate medication costs alone (without VAT) for oral methadone (100 mg/day) are less than £500 per year, £1500 per year for injectable methadone (100 mg/day), and £6500 per year using injectable diamorphine (400 mg/day) licensed in the UK [18]; with additional costs involved in dispensing and supervision. In a London trial comparing supervised injectable to oral methadone treatment, injectable treatment was found to be 4 to 5 times more expensive to deliver [19]. Given the increasing resource pressures placed upon health services, and without evidence of its cost-effectiveness over conventional treatment, IOT may be seen by many funding bodies as an unaffordable 'luxury'.

### Current evidence base for IOT

There have been several recent reviews of the evidence base for IOT [20,21]. Three published RCTs have compared injectable diamorphine to oral methadone; and one has compared injectable methadone to oral methadone.

Hartnoll and colleagues [15] reported on treatment retention and self-reported heroin use in 96 heroin users entering treatment, randomised to either take-away diamorphine ampoules (n = 44), or oral methadone (n = 52). Overall, self-reported heroin use was comparable between the two groups. Whilst the injectable diamorphine group had significantly better treatment retention at 12 months, the majority continued to use illicit heroin in small amounts. In contrast, there was greater treatment drop out in the oral methadone group, and some patients stopped using illicit heroin, whilst others continued to use larger amounts of heroin. The results did not demonstrate a clear superiority for either treatment, and it was almost 20 years before the next controlled trial.

Perneger and colleagues ([12]) reported on the first RCT of supervised injectable diamorphine, in which 51 Swiss heroin users with a history of poor performance in prior methadone programs were randomised to either injectable heroin (n = 24) or oral methadone (n = 27). Treatment retention was high in both groups, and the heroin treatment group self-reported significantly less heroin use than the methadone group. However, a considerable proportion of those randomised to oral methadone responded well (33% achieving abstinence and 19% had very low levels of heroin use), and 38% of patients randomised to the oral methadone (wait list) Control group chose *not *to enrol in diamorphine treatment when available six months later. Initiating IOT would have been unnecessary (and costly) in this patient group.

The experience from this RCT led to an understanding that IOT should be seen as a 'second line' treatment approach, generally confined to those patients who are failing to respond to their current episode of methadone treatment. This was the basis for the next RCT [14] conducted by the CCBH in the Netherlands, in which 174 methadone patients with a history of regular heroin injecting were randomly allocated to either continue their oral methadone treatment (n = 98), or to commence injectable diamorphine (n = 76) (with oral methadone doses also available). Treatment retention was comparable, and the injectable diamorphine group were reported to have had a better global response in parameters such as social functioning, psychological health and criminality.

These findings suggest that the addition of injectable diamorphine conferred benefits to patients performing poorly in methadone treatment over oral methadone treatment alone. However, there were two key limitations with the study. No data were reported for what are generally considered to be primary outcomes of treatment for heroin dependence – levels of illicit heroin use and ongoing high-risk injecting practices. We will return to this issue later. More importantly however, the study did not examine whether the addition of injectable heroin conferred benefits over 'enhanced' or 'optimal' methadone treatment. There were no specific attempts to optimise the conditions of methadone treatment for those patients randomised to the methadone only group. Hence, it may be that some patients were performing poorly at enrolment due to an inadequate methadone dose, poor psychosocial services or infrequent attendance. Without specific measures to optimize their treatment, it may not be surprising that many continued to have poor outcomes. Indeed the different outcomes between the two randomized groups could be due to the considerable differences in opioid doses used, and not due to the type or route of opioid used (the mean methadone dose in the Oral Methadone group was approximately 70 mg, whereas the mean methadone equivalent dose in the Injectable Diamorphine group was greater than 200 mg daily). It should be noted that Hartnoll et al trial [15] had a similar dose disparity (in the opposite direction, with higher equivalent oral methadone than heroin doses), thereby limiting the interpretation of two of the three published RCTs.

There are many reasons why patients may continue regular heroin injecting during their methadone treatment. These may be patient related (e.g. strong desire to continue injecting); alternatively, poor outcomes are often related to how treatment is delivered. The components of effective methadone treatment are well established [22], with more successful programs incorporating: higher doses of methadone (in particular above 80 mg); adequate levels of supervision and monitoring; access to psychosocial services; and positive therapeutic relationships between patients and service providers. In many treatment systems, optimal treatment conditions are not always available (such as access to adequate methadone doses, counselling or welfare services), and under some circumstances, continued heroin use by a patient in methadone treatment can be reduced by enhancing the conditions of their treatment – thereby diminishing the indication for IOT for that individual.

### The need for further research

The need for further controlled studies of IOT has been highlighted by a number of clinicians, researchers, user groups and political authorities (e.g. [7,19,23,24]). Systematic reviews of the evidence regarding IOT have concluded (a) the provision of IOT in supervised injecting clinics is feasible, (b) that IOT appears to be at least as effective as conventional substitution treatment in achieving certain outcomes such as treatment retention; however (c) the existing evidence base is insufficient: "No definitive conclusions about the overall effectiveness of heroin prescription is possible because of non-comparability of the experimental studies available. Heroin use in clinical practice is still a matter of research in most countries" [20]. We note that there are several RCTs of injectable heroin treatment that have been recently conducted or in progress (in Germany, Spain and Canada), and the findings of these trials will substantially build upon the available evidence base.

Given the concerns regarding IOT and its relatively limited evidence base, the principles of rational therapeutics would suggest that IOT should be seen as a 'second line' treatment modality limited to patients in methadone treatment who meet the following criteria: (i) have a protracted history of heroin dependence and injecting, (ii) have adhered to conditions of effective methadone treatment for an extended period of time, yet (iii) continue to regularly inject illicit heroin and experience related harms [25]. To date however, no controlled trial of this target group has adequately examined whether the prescribing of injectable opioids is more effective than attempts at enhancing the conditions of oral methadone treatment in those patients performing poorly in their current treatment episode.

Further, the social and historical context in which treatment occurs must be considered. Recent guidance [25] emphasises that all patients entering IOT should have full supervised dosing (as with patients commencing oral substitution treatment), through the establishment of new 'European-style' clinical services. This is a new development for UK services, and it may be that the feasibility of delivering supervised IOT in Swiss, Dutch or German cities may be difficult to replicate in Britain, where there may be different patterns of drug use, geo-demographics, and importantly, different expectations among service users of IOT – given the long tradition of unsupervised IOT in this country.

Of particular relevance to the British setting is the limited evaluation of injectable methadone treatment. The majority of IOT in the UK is in the form of injectable methadone, and yet there has been only one RCT of injectable methadone, a pilot study in which 40 heroin users entering treatment were randomly allocated to either equivalent doses of oral methadone or injectable methadone. There was no significant difference in treatment retention, illicit heroin use or other outcomes between the two groups [19]. The use of injectable methadone rather than diamorphine may have particular advantages (e.g. only one daily injection, less expensive) or disadvantages (e.g. side effects, inadequate substitute for heroin). Further research is required to establish the role of different injectable opioids.

Another concern in the interpretation of earlier research has been the reliance on self-report data in evaluating illicit heroin use. The research examining the validity and reliability of self-reported heroin use (see [26] for review) indicates that "self-report of illicit behaviours are sufficiently reliable and valid to provide descriptions of drug use, drug-related problems and the natural history of drug use" (p. 261–262). However, the conditions required to achieve this include that (a) there are no negatively perceived consequences for the client arising from any self-disclosure (such as loss of take-away privileges, loss of face with a therapist or researcher, or even discontinuation of the program); and (b) self-report coincides with, and can be corroborated by, objective measures such as urine drug screens. Hence, the evaluation of illicit heroin use in opioid substitution treatment trials normally involves the confidential collection of self-report data by independent researchers, together with regular urine drug screens.

It is difficult to achieve these conditions when evaluating diamorphine treatment. The detection of morphine in urine drug screens (UDS) may be a suitable marker for heroin use in methadone patients, but is useless as a marker in diamorphine prescribed patients. Further, the possible perception by some clients that unfavourable outcomes for a trial-based diamorphine program could result in its discontinuation or scaling back, could serve as a motive for clients to under-report their illicit heroin use, particularly in the absence of UDS. Under such circumstances, the validity and reliability of self-reported illicit heroin use may be questioned. In response, there have been efforts to establish objective measures of illicit heroin use that do not rely on the detection of morphine. A promising technique includes the identification of papaverine metabolites in UDS – papaverine is an opiate found in illicit 'brown' heroin commonly used in western Europe, but not present in pharmaceutical diamorphine [27,28]. Recent research suggests papaverine metabolites (hydroxy- and dihydroxypapaverine) are a suitable marker for illicit heroin use, with high sensitivity, specificity and negative predictive values compared to the detection of morphine in UDS of methadone or buprenorphine prescribed patients [28]. This approach provides an avenue for objective assessment of illicit heroin use in diamorphine-prescribed patients.

The development of the Randomised Injectable Opioid Treatment Trial occurred as a response to calls from the Home Office to expand IOT in Britain [24], the need to better establish an evidence base for IOT, and to examine the role of injectable methadone and diamorphine treatment delivered under the conditions identified in new UK national guidance [25]. Specifically, the central question to be addressed is whether efforts should be made to optimise conventional treatment for such patients (e.g. encouraging high doses, supervised dosing, psychosocial interventions, and regular attendance) in order to reduce regular illicit heroin use, or whether such patients should be treated with injected methadone or injected heroin in newly developed supervised injecting clinics. To date, this has not been addressed in published RCTs of IOT.

The research component of the trial was primarily funded by a Research Grant from Action on Addiction, a national voluntary sector charity and the Big Lottery. Clinical services (including the establishment of new supervised injecting clinics) were funded by the Home Office and National Treatment Agency, together with local health authority funding. The research is being conducted by researchers at the National Addiction Centre (Institute of Psychiatry, Kings College London and the South London and Maudsley NHS Trust).

## Methodology

### Overview

**Figure 1:**
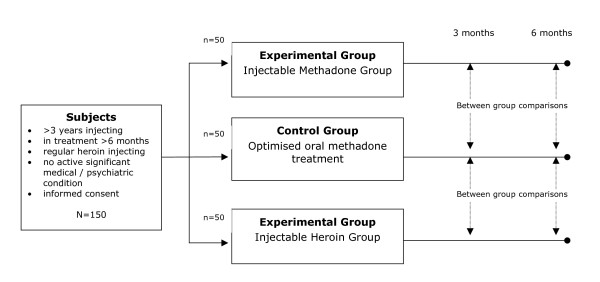
Overview of Research Design for RIOTT.

RIOTT examines the role of IOT for the management of heroin dependence in patients not responding to conventional substitution maintenance treatment. The study is a prospective, open-label three-way RCT (See figure 1). Eligible patients (in oral methadone treatment but still injecting illicit heroin on a regular basis) are randomised to one of three conditions: (i) optimised oral methadone treatment (Control group); (ii) injected methadone treatment; or (iii) injected heroin treatment. Approximately 150 subjects (50 in each group) are followed up for 6-months, comparing between-group differences on an intention-to-treat analysis between the Control Group and the Injectable Methadone Group, and between the Control Group and the Injectable Heroin Group; comparing outcomes across a range of measures, including drug use, injecting practices, measures of global health and psychosocial functioning, criminality, treatment retention, incremental cost effectiveness, and measures of client satisfaction. It is a multi-site trial conducted at four sites in London (two sites), Brighton (South East England), and Darlington (North East England).

### Research hypotheses

The aim of the study is to examine the safety, efficacy and cost effectiveness of treatment with optimised oral methadone compared to injectable methadone or injectable diamorphine, for patients in maintenance treatment who continue to inject illicit heroin regularly. The trial is not designed to directly compare injectable methadone to injectable diamorphine treatment, which are both 'experimental' conditions. Hence, the research hypotheses for injectable heroin treatment are:

i) that a selected group of patients (not responding to current oral methadone treatment) receiving injectable heroin treatment, will make greater reductions in their illicit heroin use, other drug use and criminal activity, and greater improvements in their health and social functioning, than if provided with optimised oral methadone treatment;

ii) providing injectable heroin to a selected group of patients (not responding to current oral methadone treatment) results in a greater economic benefit per extra unit of resource invested in the treatment, than only offering optimised oral methadone;

iii) The formal null hypothesis is that: there is no difference between the two treatments

The research hypotheses for injectable methadone treatment are:

i) A selected group of patients (not responding to current oral methadone treatment) receiving injectable methadone treatment, will make greater reductions in their illicit heroin use, other drug use and criminal activity and greater improvements in their health and social functioning, than if provided with optimised oral methadone treatment;

ii) providing injectable methadone to a selected group of patients (not responding to current oral methadone treatment) results in a greater economic benefit per extra unit of resource invested in the treatment, than only offering optimised oral methadone;

iii) The formal null hypothesis is that: there is no difference between the two treatments

The primary outcome measure is illicit heroin use as measured by the proportion of subjects in each group who cease *regular *illicit heroin use, operationalised as providing at least 50% UDS negative for markers of illicit heroin during months 4 to 6 of treatment (allowing a sufficient period for the study treatment to take effect). We estimate at least 50% negative UDS in once weekly random UDS to be consistent with no more than approximately one (or at most two) days heroin use per week on average – which represents a discontinuation of *regular *heroin use, and is a clinically meaningful reduction for this particular patient population (selection criteria include using illicit heroin on at least 50% of days and 100% positive UDS screens for heroin use). The full range of measures for illicit heroin use is described in the Outcome Measures section below.

The secondary outcome measures are:

- other illicit drug and alcohol use,

- high risk injecting practices and complications,

- indices of general health and psychosocial functioning,

- criminal activity,

- treatment retention,

- adverse events,

- cost-effectiveness in enhancing quality of life indices and reducing illicit heroin use.

- patient satisfaction with each of the three treatment approaches

- treatment goals and priorities of patients entering the trial;

- likely demand for any future expansion of IOT under these conditions.

### Subjects

#### Selection criteria

The trial targets patients in methadone treatment who continue to inject heroin on a regular basis. Specific criteria are:

1. Aged between 18 and 65 years at recruitment to study.

2. At least 3-year history of injecting heroin use.

3. In continuous methadone treatment for at least 6 months this episode.

4. Regular injecting heroin use in preceding 3 months (as evidenced by opiate-positive urine drug screens and self-report in clinical records), and heroin use on at least 50% of days (15 days) in the preceding month on self-report.

5. Evidence of regular injecting on clinical examination.

6. No significant and active medical (e.g. hepatic failure) or psychiatric condition (e.g. active psychosis, severe affective disorder).

7. Not alcohol dependent or regularly abusing benzodiazepines according to DSM-IVR criteria.

8. Not pregnant, breastfeeding, or planning to become pregnant during the study period.

9. Resident of catchment area of participating agency.

10. Able and willing to participate in the study procedures (e.g. no impending prison sentence) and provide informed consent.

#### Sample and power calculation

Primary statistical comparisons will be made between the Control and Injectable Methadone groups; and between the Control and Injectable Heroin groups. The primary endpoint for the sample size calculation is the proportion of subjects in each group who cease regular illicit heroin use (operationalised as providing at least 50% UDS negative for markers of illicit heroin) during months 4 to 6 of treatment – allowing a sufficient period for the study treatment to take effect.

Due to the differences in selection criteria and reported outcome measures in previous studies of IOT, the available evidence does not allow confident predictions of treatment effect size for the different conditions. Nevertheless, some estimates can be made based on earlier trials [12,14,19,29]. Assuming 20% of subjects in the optimized oral methadone group cease regular illicit heroin use (as operationalised above), and 50% of patients in each of the IOT groups cease regular illicit heroin use, using a three-way randomisation schedule with equal numbers assigned to each group, and with α = 0.05, β = 0.85, then approximately 50 subjects per group should be sufficient to detect significant differences between the Control group and each of the Experimental Injectable groups – 150 subjects in total.

#### Recruitment and treatment allocation

Information regarding the trial is made available to patients in participating catchment areas through a variety of means, including written information at clinics, existing service providers, and through service user group meetings. Patients receiving oral methadone treatment who inject illicit heroin regularly may refer themselves, or be referred by their keyworker to the RIOTT clinical team at each site. Those identified as potentially eligible are assessed and screened by RIOTT clinical staff to establish eligibility and informed consent. The assessment, screening and consent process usually takes approximately two weeks from initial presentation.

Randomisation for the trial is conducted independently by the Clinical Trials Unit of the Institute of Psychiatry, King's College London. Subjects are randomly allocated using minimization techniques into one of the three study conditions in a 1:1:1 ratio within each treatment site, with stratification on two criteria: a) regular cocaine/crack use (> 50% days in previous 4 weeks on self-report); and b) receiving optimised oral methadone treatment at baseline (doses of at least 80 mg/day and supervised at least 5 days/week).

Randomisation occurs at the end of the two-week assessment and consent period. Patients receive pre- and post-randomization counseling by the clinical team prior to being informed of their treatment allocation by a researcher. Treatment is initiated on the next working day.

### Treatment conditions

The study is an open-label trial in which clients, clinicians and researchers are aware of treatment allocation. The same clinical staff deliver all three treatment conditions at each site, thereby minimizing the potential for bias as a result of differences in staff expertise or enthusiasm. The treatment conditions are:

#### (1) Optimised oral methadone treatment

Patients randomised to the Control group are to receive 'optimised oral methadone treatment'. The key principles for treatment in this group entail:

- *High methadone doses*. Doses are individualised with the aim of reducing illicit opiate use. Methadone doses in excess of 80 mg (and generally >100 mg) are encouraged (but not mandated) in this patient group, with a maximum upper dose of 300 mg identified.

- *Supervised dosing: *Oral methadone is consumed under supervision on at least 5 days per week during the first 3 months. Thereafter, the level of supervised dosing may reduce (conditional upon the patient's substance use, medical, psychiatric and social circumstances) to three days a week, which is the minimum attendance required to enable random urine collection for drug screening.

- *Frequent reviews and ancillary services*. Patients are assigned a key worker, with weekly sessions scheduled during the initial three-month period. Thereafter, the frequency of scheduled reviews may reduce to two-weekly. All patients have monthly reviews with a study medical officer, and have access to a psychologist for individual CBT-based therapy. Patients also have access to other ancillary services (e.g. group programs) available at each site, as for any patient of the service. It should be emphasized that participation in ancillary services such as counseling or group activities are voluntary, and not a requirement of continuation in the study.

- *Treatment Care Plans *addressing drug-related, physical, psychological and social issues are developed in consultation with the client, key worker, study medical officer, and other relevant parties within the first month of the study, and reviewed at 3 and 6-months. Other health or social services are engaged accordingly.

- *Urine drug screens*. Random urine drug screens collected weekly.

#### (2) Treatment with Injectable Methadone or (3) Injectable Diamorphine

Aspects of treatment other than medication issues (such as key worker and medical reviews, ancillary services, and urine drug screens) are the same for the Injectable Methadone and Injectable Diamorphine groups as described for the Optimised Oral Methadone Group.

- *High doses of injectable methadone or diamorphine*. For the Injectable Methadone group, initial doses are converted from oral to injectable methadone. Whilst there is limited evidence regarding conversions between injected and oral methadone in this population, the available data suggests that oral methadone has a mean bioavailability of approximately 80%, with large individual variation (ranging from 40 to 100% in previous research) [30-33]. Hence, the study uses a conversion formula of injected methadone dose = 0.8 × oral methadone dose (separate research underway at the National Addiction Centre is examining the biavailaibility of injectable methadone (IM and IV) in long term oral methadone patients). Doses are subsequently titrated (generally upwards) and individualised with the aim of reducing illicit opiate use. Patients can also choose to have oral methadone supplements. Maximum doses of injectable methadone are 200 mg per day (plus up to 100 mg oral methadone), to a total dose of 300 mg per day.

Injectable Diamorphine group: the dose conversions between oral methadone and injected diamorphine are based upon the work of Seidenberg and colleagues [34], developed for the Swiss, and more recently used by the German and Canadian heroin trials. The dose equivalence between oral methadone and diamorphine is not linear. At low doses, the conversion rate from oral methadone (total daily dose) to injected heroin (total daily dose) is approximately 1:3; whilst at higher doses, the conversion rate approximates 1:5. Other factors that impact upon methadone metabolism (e.g. concomitant medications, medical conditions) are taken into consideration at transfer. Doses are subsequently titrated and individualised with the aim of reducing illicit opiate use. Patients are encouraged to retain a small oral methadone dose (e.g. 20 to 40% of their initial dose) in order to prevent opiate withdrawal between injecting sessions, and to facilitate any transitions between oral methadone and injected diamorphine (effectively having a 'loading dose' of methadone). It is expected that most patients will use injected diamorphine doses in the range of 300 to 600 mg per day, with an upper total daily dose of 900 mg (450 mg per injection). Patients can also have up to 100 mg oral methadone supplementary to diamorphine, making their total oral methadone equivalent dose approximately 300 mg.

- *Supervised dosing*. All doses of prescribed injectable opioids are supervised throughout the 6-month study period. Treatment typically involves once-a-day injection of methadone, or twice-a-day injection of diamorphine. Patients have a degree of autonomy in the frequency of attendance for dosing and the mix of injected opioids/oral methadone – patients unable or unwilling to attend for injectable opioid treatment have access to oral methadone doses. This flexibility of attendance for on-site injecting aims to minimize the inconvenience of IOT, and reflects that many patients entering RIOTT may not be injecting every day, and hence, it may not be therapeutically necessary for patients to increase their frequency of injecting. An example of this dosing flexibility is provided in Table 1. Patients must attend a minimum of 4-days-a-week for onsite IOT (to ensure integrity of the treatment condition). The principles of IOT used in RIOTT are consistent with recent national guidance [25].

**Table 1:** Example of flexibility in IOT prescription

	Drug & route	Dose & time
Regime A	Diamorphine (IV or IM) Diamorphine (IV or IM) Methadone (oral)	200 mg morning 200 mg afternoon 30 mg evening
Regime B	Diamorphine (IV or IM) Methadone (oral)	200 mg (daily) 100 mg (daily)
Regime C	Methadone (oral)	160 mg

All doses of injectable opioids are supervised onsite in the participating clinics. Two injecting sessions operate each day, 7-days a week. Patients self-administer their injections, with the choice of intravenous, intramuscular or subcutaneous routes. Injecting sites and routes are recorded daily, and routinely assessed throughout the trial.

#### Medications

Trial medications include (i) oral methadone solution (1 mg in 1 ml) or concentrate (10 mg in 1 ml); (ii) injected methadone ampoules (50 mg in 1 ml, 50 mg in 2 mls, 10 mg in 1 ml ampoules licensed in the UK for IM or IV injection); and (iii) diamorphine: the trial uses 10 gram freeze-dried diamorphine ampoules licensed and imported from Switzerland (Diaphin^®^), which are reconstituted by a trial pharmacist under aseptic conditions to a concentration of 100 mg/1 ml. Each injection of diamorphine is dispensed as a 'loaded' syringe by the pharmacist, and self-injected by the patient. The trial has a Clinical Trial Authorisation from the UK Medicine and Health Regulatory Authority for importation and use of Diaphin^® ^ampoules.

#### Treatment post-trial

As injectable methadone and injectable diamorphine are licensed and available in Britain, the trial does not need to consider issues of 'compassionate grounds' for continuation of treatment. At the end of the 6-month study period, the nature of ongoing treatment is decided on an individual basis by clinicians in consultation with each patient, in keeping with the recent NTA Guidance Report [25], and subject to available clinical resources. The basis of this decision will be the extent to which each patient has demonstrated a positive clinical response and has obtained significant benefit from their study treatment.

### Outcome measures and data collection

The study utilises a combination of data collection methods for the outcome domains, including self-report data, UDS, and clinical records. Structured interviews with subjects are conducted by independent (IoP) researchers at baseline (during the pre-trial treatment phase), 3 and 6 months after randomisation. Semi-structured interviews are conducted at various time points over the trial.

#### Efficacy

The primary outcome for the trial is illicit heroin use, measured using self-report data and UDS results, using papaverine metabolites as markers of illicit heroin use [28]. The secondary outcomes include changes in other types of substance use, high-risk injecting practices, indices of general health and psychosocial functioning, criminal activity, treatment retention, and indices of patient satisfaction. Outcome measures are described in Table 2.

**Table 2:** Outcome measures

*Outcome*	*Outcome measures*
Illicit heroin use	• Self-reported data at 3 and 6 month interviews (including number of days used, routes of administration, average amount/cost, and frequency of use in past month as measured by the *Opiate Treatment Index *(OTI) Q score [43]; self-reported overdoses. • Random weekly UDS result, testing for papaverine metabolites.
Other drug use	• Self-reported data at 3 and 6 month interviews regarding use of other opioids, alcohol, benzodiazepines, cannabis, cocaine. Measures include number days used, average cost/amount used. • Random weekly UDS result
High-risk injecting practices	• Self-report data at 3 and 6 month interviews regarding participation in risk practices for blood borne virus transmission in preceding month using modified *Injecting Risk Questionnaire *[44] • Self-report data regarding injecting practices in past month (including sites, routes, adverse events, complications) and clinical examination of injecting sites (monthly medical reviews)
General health status and psychosocial functioning	• Self-report data using *SF-36 *[45], *EQ-5D *[46, 47] and *OTI Psychosocial Adjustment Section *collected at 3 and 6 month interview • *Hospital Anxiety Depression Scale *[48] completed at monthly medical review.
Changes in criminality	• Self – report data using modified Crime Section of *Maudsley Addiction Profile *[49] and OTI collected at 3 and 6 month interview
Measures of patient expectation and satisfaction	• *Treatment Perceptions Questionnaire *[50] • *Drug Use Expectations *and *User Nominated Outcome Instrument *structured and semi-structured interviews examining patient perceptions of positives and negatives of using illicit heroin, and key outcomes/goals of treatment, as identified by service users (developed for the trial). Interviews conducted with service-user researcher at baseline, 3 and 6 months • Semi-structured interviews with service-user researcher examining patient perspectives of the relative advantages and disadvantages of each of the three treatment approaches.

#### Safety

Adverse events to injected prescription heroin have been described in Swiss heroin clinics [35], however further information comparing the adverse profile of injected diamorphine, oral and injected methadone in chronic addict populations is needed to better characterise the safety profile of these medications. Adverse events may occur as a result of the drug (e.g. methadone, heroin), or the route of administration (IV, IM, oral). Indeed, anecdotal reports suggest that some patients experience considerable adverse reactions (e.g. pain, 'burning') with intravenous methadone, however these have not been adequately documented in the published literature. RIOTT will examine adverse events between the three treatment groups over time.

There are also potential concerns regarding the cardio-respiratory effects of injectable opioids. Previous studies examining self-administration of injectable heroin and methadone have identified significant hypoxia in some patients [36,37]. However, it is unclear to what extent the risk of hypoxia is influenced by treatment conditions (e.g., injected drug, route of administration, dose). There have also been recent concerns regarding the use of high dose (oral and injected) methadone and prolongation of QTc intervals, a condition associated with cardiac arrythmias (such as torsade de pointes) and sudden death [38,39]. It remains unclear whether injected methadone poses a greater risk of QTc prolongation than equivalent doses of oral methadone. The acute effects of injected diamorphine upon ECG changes have not been previously reported. To address these concerns, indices of respiratory and cardiac function are examined in relation to the drugs used (methadone versus diamorphine), route of administration (oral, IV and IM), and dose, compared to pre-randomisation baseline measures.

The prescription and supervised injection of diamorphine (and methadone) allows the opportunity to study the physiological, subjective and cognitive-performance effects of these drugs. Despite widespread use of injected heroin in many societies, there is to date remarkably little published literature about the impact of injected heroin or methadone upon these parameters. The capacity to study the effects of heroin under clinical/laboratory conditions will enhance our understanding of the acute effects of heroin use in dependent users.

Another aspect of safety of this treatment approach is that of 'community safety'. One of the major issues facing those providing drug treatment services is the possible community backlash when such services are proposed within a local community. The establishment of a supervised injecting clinic raises potential concerns for the local community. Examples may include fears of local residents and businesses regarding the congregation of drug users regularly attending the clinic; or concerns regarding intoxicated clients being a nuisance to the local community. As a part of the overall RIOTT, a community impact evaluation will be conducted to: a) investigate the impact on the local community of supervised injectable maintenance clinic; b) document the expectations, fears and experience of the local community; and c) compare and contrast the methods used by different services for addressing community interaction. The study design incorporates a blend of epidemiological and social research methodologies in order to gather data from a number of complimentary sources.

#### Cost effectiveness

IOT is likely to be more expensive than optimised oral methadone treatment, yet may be associated with better outcomes and/or cost-savings elsewhere. The economic evaluation will take a broad cost perspective, including costs borne by the health, social, voluntary and criminal justice sectors, and costs to the economy in the form of productivity losses. Detailed information on the resources associated with the three treatment interventions are collected from the relevant clinics and include staff time, equipment, study medications, dispensing services and the treatment of adverse events. Data on the use of all other services (including health, social and voluntary sector services), days off work due to illness, criminal justice sector contacts and crimes committed are collected using a service use schedule, based on one designed at the University of York for the economic evaluation of alcohol and drug interventions and successfully applied to evaluations of brief interventions [40]. Self-report data are collected at research interviews at baseline and at the 3 and 6 month follow-up points. Cost-effectiveness will be explored in terms of illicit heroin use, the primary outcome measure, and in terms of quality adjusted life years, using the EQ-5D measure of health-related quality of life (see Table 2).

#### Patient satisfaction and experiences

A service-user researcher (CH) employed through a service user-organisation, the Alliance, conducts structured and semi-structured interviews with subjects throughout the follow-up period to gain information on expectations of, and satisfaction with treatment (see Table 2). Semi-structured interviews will also explore the broader experience of prescribed and non-prescribed heroin use, exploring the contextual influence of the clinical setting itself in the construction of individual's experiences of heroin use. It is expected that interviews with a service user researcher may lend greater validity to subject responses when examining issues such as treatment goals, perceptions about illicit heroin use and treatment.

#### Data analysis

Quantitative data will be recorded by hand in Case Record Forms, coded, and entered into a database using the Statistical Package for Social Science-12 software by a researcher. Data will be analyzed on an intention-to-treat basis. The primary outcome measure (illicit heroin use – operationalised as the proportion of subjects who provide >50% UDS negative for markers of illicit heroin use during the 4^th ^to 6^th ^months of study treatment) will be analysed using the logistic regression model. Continuous outcomes collected at 3 time points (baseline, 3 months and 6 months follow-ups), will be modelled using repeated measurement with mixed models approach to examine the differences between treatment and control groups and the time course. Correlated responses will be dealt with by covariance structures. Endpoints that are coded as categorical data will be treated as multivariate binary responses in order to fit the repeated measurements. Odds ratios will be used to present differences in treatment retention between the control and IOT groups.

Semi-structured interviews collecting qualitative data are tape recorded (with subject consent) and transcribed, with all identifying information removed from the transcripts. Data is analysed in hard copy form, using qualitative thematic analysis [41].

## Discussion

Despite IOT being part of the British treatment system, there have been few studies examining the safety and effectiveness of this approach, particularly of injectable methadone treatment, which comprises the majority of IOT in this country. The primary aim of RIOTT is to compare the safety, efficacy and cost effectiveness of injectable methadone and diamorphine treatment (delivered under supervised conditions), to optimised oral methadone treatment, in methadone patients who regularly inject illicit heroin. Such research should better inform clinicians and policy makers as to how to respond to this patient group, and to inform decision making as to whether additional resources should be directed to IOT, or to enhancing the capacity for oral methadone treatment to be delivered under optimised conditions.

The establishment of RIOTT also enables a variety of 'nested' studies examining a range of issues regarding injectable heroin and methadone. Some of these studies have been briefly discussed, such as projects examining the cardio-respiratory effects of these drugs in chronic opiate dependent individuals. Other projects being examined include cognitive-performance effects of injectable opioids compared to oral methadone, and pharmaco-genetic research examining responses to opioid treatment. Despite the widespread proliferation of heroin use across the world, and despite reports of widespread misuse of methadone by injection [42], there has been remarkably little laboratory research examining the pharmacological effects of these drugs at high doses in chronic opiate users. Such research may lead to a better understanding of the mechanisms behind heroin-related harms such as cognitive impairment and overdose.

A key point in embarking on another RCT of injectable heroin treatment relates to the extent to which there is a sufficient evidence base currently available to scientifically 'prove' the efficacy of heroin prescribing – in short – do we need more heroin trials? Underlying this is the question 'how much evidence is required in order to scientifically establish the efficacy and safety of a particular treatment'? The experience of substitution opiate treatment (methadone, LAAM, buprenorphine) suggests that considerable and unequivocal evidence is required in order to defend such treatments against politicised critics – be they politicians, academics, service providers, local community or (ex)drug user groups – much more so than in most other areas of medicine. This applies to an even greater extent with the highly controversial issue of injectable opioid prescribing. A particularly strong evidence base will be a minimum requirement in establishing IOT as an available option – the findings of two or three RCTs alone are unlikely to convince sceptics of such a contentious treatment paradigm. The most recent systematic Cochrane review of heroin prescribing suggests that differences in study design limit our capacity to draw conclusions on the available published trials in this area. The completion and dissemination of the various international trials in Germany, Spain, Canada and Britain is required in order to establish this evidence base, particularly as we should not pre-empt the findings of such studies in assuming that they will all show similar results. Should the broad safety and efficacy of IOT be established through the various trials underway internationally, it will enable researchers to address the next 'level' of clinical-research questions. This may include issues around the availability of take-away doses of injectables, the selection of which opioids should be prioritised, and whether patients can be successfully transitioned from injecting to non-injecting (e.g. intranasal, oral) routes.

We should also recognise that, as with the expansion of methadone and buprenorphine treatment internationally over the past 20 years, any expansion of IOT will often be preceded by calls for 'local' research that addresses local concerns and regulatory requirements. Interestingly, whilst Phase III RCTs are often conducted in order to achieve registration of a new medicine, in the British context however, licensing of IOT is not the issue. Rather research is required to establish its role within the treatment system after over a decade of decline, and at a time where health systems are requiring a greater evidence base of efficacy and cost-effectiveness.

## Competing interests

The author(s) declare that they have no competing interests.

## Authors' contributions

NL and JS are the Chief Investigators, and NM is the Trial Co-ordinator and Co-investigator. These three authors have equally contributed to the drafting of this report. SB, SL, and CH are the health economist, statistician and service user researcher for the trial and have contributed to the relevant sections of the paper. All authors read and approved the final manuscript.
